# Objectively measured physical activity and sedentary time in south Asian women: a cross-sectional study

**DOI:** 10.1186/1471-2458-14-1269

**Published:** 2014-12-13

**Authors:** Whitney B Curry, Janice L Thompson

**Affiliations:** School of Health, Sport and Bioscience, University of East London, London, E15 4LZ UK; School of Sport, Exercise & Rehabilitation Sciences, University of Birmingham, Birmingham, B15 2TT UK

**Keywords:** Accelerometry, Measurement, Moderate-to-vigorous physical activity, Activity patterns

## Abstract

**Background:**

South Asian (SA) women in the United Kingdom (UK) are known to be at high risk for developing chronic diseases, and to have low levels of physical activity (PA). Increasing levels of PA and reducing sedentary time (ST) are recognized as factors to target in an effort to curb chronic disease morbidity and mortality. There is limited evidence documenting objectively measured PA/ST and their correlates in SA women. Therefore the purpose of this cross-sectional study was to objectively measure and report patterns of PA/ST among SA women in the UK and examine potential socio-demographic correlates of PA/ST.

**Methods:**

140 UK SA women (Pakistani and Bangladeshi) wore an accelerometer for 7 consecutive days. Anthropometric measurements and self-reported socio-demographic information were taken.

**Results:**

Mean daily moderate-to-vigorous PA (MVPA) was 34.66 ± 21.52 minutes and mean daily ST was 530.20 ± 81.76 minutes, with an inverse correlation (*r =* -.270, N = 140, p < .001) showing that higher ST was associated with lower MVPA. The same was seen for light intensity PA (LPA) (*r =* -.610, N = 140, p < .001). MVPA was significantly higher for younger women (18–64.5 yrs), with 64.7% of older women (≥65 yrs) failing to meet PA guidelines (*t =* 3.101, p < 0.05). Overweight/obese women had lower levels of LPA, MVPA and higher ST (p < .001). Multiple linear regression analyses indicated 14.9% of the variance in MVPA was explained by age and waist circumference (F(2,138) = 6.41, *p <* 0.002). LPA and ST were significantly higher on weekend days than weekdays (p < .001), and MVPA was significantly higher on weekdays than weekend days.

**Conclusions:**

Results indicate higher levels of PA in this sample than previously assumed. High levels of LPA in this sample indicate the need for health promotion interventions to target not only duration, but also intensity of activity in this population to achieve PA recommendations.

## Background

Low levels of physical activity (PA), defined as energy expenditure produced by any skeletal muscle bodily movement [1], and increased sedentary time (ST), defined as time spent engaging in sedentary behaviors including those that expend ≤1.5 METS while sitting or laying [2], are recognized as key independent risk factors in the development of cardiovascular disease (CVD) and are significant contributors to other chronic conditions such as type 2 diabetes and obesity [3, 4]. A substantial body of evidence has accumulated which supports the causal relationship between increased PA with decreased risk for CVD, with over 24 prospective cohort studies between 1997–2008 indicating this relationship [5]. It has been shown that those who are physically active can reduce their risk of developing CVD by nearly 50% [6]. Ethnic minority groups, women, and those of lower socioeconomic status in the UK are known to have higher morbidity and premature mortality from CVD [7] and other chronic diseases than white European populations [7]. SA women in the UK are a group known to be affected by all of these factors [3, 8]. In the UK, 33% of all mortality in SA in 2008 was caused by CVD [6].

Major health organizations currently recommend engaging in 150 minutes of moderate intensity PA, or 75 minutes of vigorous activity, per week to achieve health benefits [9–11]. Emerging evidence suggests that decreasing ST may also decrease risk for CVD [6]. A recent review of prospective studies examining ST indicated increased ST was associated with increased risk of fatal and non-fatal CVD [12]. Self-report data from the Health Survey for England indicate that Bangladeshi and Pakistani women in the UK are less likely to meet PA recommendations and are more sedentary than their white counterparts [13], and are less likely to participate in regular sporting activity [14]. Therefore, PA/ST are potentially modifiable health behaviors that can be targeted for behavior change to reduce risks for morbidity and premature mortality resulting from various chronic diseases. Sex, age, socio-economic status, BMI, waist circumference, and body fat percentage are commonly thought to be determinants of PA, and are being increasingly explored in relation to ST [14]. Additionally, English language ability and migration status are now considered variables requiring exploration due to how they may influence the ways in which people access services and engage in various forms of PA [15].

It is important to accurately assess PA and ST in all populations in order to enhance surveillance and examine trends, and to develop and evaluate appropriate and effective prevention and intervention strategies to increase PA and reduce ST [16]. There is currently no generally accepted standardized method of accurately assessing PA and ST, although self-report questionnaires and objective methods such as accelerometry are now widely used [17]. To date, there is limited published research reporting PA/ST levels in SA women and few studies investigating activity levels of older SA women, a group that may be at risk for low activity levels and high ST [17, 18]. A systematic review investigating PA/ST among SA women (aged 16 to 90 + yrs) found that the existing published evidence is of relatively low quality [17]. There is no published evidence of the validity of self-report methods assessing PA/ST in this group. Only 2 studies examined ST using self-report measures [4, 19]. This review highlighted that self-report data indicate low levels of PA in SA women when compared to SA men and white adults [17].

Accelerometers are a common method of objectively measuring PA/ST due to their convenience and durability [20]. They are small motion sensors that record frequency, intensity and duration of PA and can detect ST [20]. Accelerometers can detect activity in a free-living environment and are practical for measuring large groups [20]. There is some evidence that accelerometers can be used to collect PA/ST data on groups who may have limited English language skills and/or low literacy levels, as may be the case amongst some SA women in the UK [19]. Only two studies have objectively measured PA in this population [17]. One study that examined PA among SA was based in New Zealand and was conducted on Asian Indian adults. Participants wore a pedometer to objectively measure PA, with PA levels classified based on step counts [21]. The authors reported that 33% of their sample (n = 112) were active (defined as accruing more than 10,000 steps per day) [21]. The other study examining PA using objective measurement employed the Caltrac accelerometer (PA reported in Kcal/day) among Asian Indians living in the UK and India [22]. Findings indicated that Asian men and women in India were more active than those in the UK [22]. To date, there appear to be no published studies that have objectively measured ST in this group. Due to the low quality of self-report data, the extreme lack of objectively measured PA/ST data on SA women, including Bangladeshi and Pakistani women, and absence of ST data almost entirely, more research using objective measures is needed to investigate levels and patterns of PA and ST among SA women.

Thus, the aim of this cross-sectional study is to objectively measure and report the amount and patterns of PA/ST among Pakistani and Bangladeshi women (referred to as SA women for the purposes of this study) in Cardiff, Wales, examine potential socio-demographic correlates of PA/ST in this group, and to make recommendations for future research in this area.

## Methods

### Study population

This convenience sample consisted of Bangladeshi and Pakistani women aged 18–72 years from Cardiff, Wales, and is thus not representative of the general population of UK SA women. The majority of research amongst Bangladeshis in the UK has concentrated on the Tower Hamlets region of London, limiting the amount of knowledge on those communities living outside of the London area [23]. The majority of research on Pakistanis in the UK has been conducted in the Manchester and Bradford areas [24–27]. Findings from existing studies may have limited generalizability to other communities in the UK; therefore more research is needed to expand our understanding of these minority ethnic groups and how to improve their health and well-being throughout the UK.

Cardiff has approximately 25 distinct ethnic minorities making up over 10% of its population [28]. In 2011 it was estimated that those of SA descent made up 4% of the population, with nearly 4,838 Bangladeshis and 2,637 Pakistanis living in Cardiff [28]. While there was a great deal of migration of adults from Bangladesh and Pakistan to the UK between 1950 and 1980, there is now an increasing number of individuals within these groups who were either born in the UK or moved to the UK during childhood [23, 29]. Bangladeshi and Pakistanis in the UK are relatively young, with 4.2% over the age of 65 as compared to 7.2% of the White population in the UK older than 65, based on the 2011 census data [30].

Recruitment took place from January 2012 through January 2013, and built upon a sample initially recruited from another study conducted in Cardiff [31]. The full sample was obtained through referrals and contacts with community groups in the local areas. Participants were eligible to participate if they were a woman 18 years or older, born in Bangladesh or Pakistan and currently residing in the UK, or born in the UK to Pakistani or Bangladeshi parents and currently residing in the UK, ambulatory, had no medical reason that prohibited participation, and were able to give informed consent. Written and verbal consent were obtained from respondents. The ethical review committee of the University of Birmingham approved this study (reference # ERN_12-1316).

### Measures

Descriptive data included height (to the nearest mm), weight (to the nearest 0.1 kg), waist circumference (to the nearest cm), and body fat percentage measured using the Bodystat Quadscan 4000 (BodyStat Ltd, Douglas, Isle of Man, British Isles) estimated to the nearest 0.1% using an equation validated with SA adults [21]. Age, current health/disease status, medications, place of birth as an indicator of migration status, current residential postcode in the UK, and self-reported English language fluency were self-reported by questionnaire. BMI was calculated by dividing weight in kilograms by the square of height in meters. The Welsh Index of Multiple Deprivation (IMD) was used as a socioeconomic indicator and was determined based on residential postcodes.

PA/ST was assessed using the ActiGraph GT1M and GT3X accelerometers (ActiGraph, Pensacola,FL). It has been reported that there is no significant difference in measurement between the two accelerometer models [20]. Therefore no additional calibration or validation studies were done to compare the data derived from the models. Accelerometers were programmed to record activity data in 60-second epochs. Participants were asked to wear the monitor during waking hours for 7 consecutive days, removing it to sleep or for water-based activities such as bathing or swimming. Previous research suggests that removal of the device for water-based activities should not underestimate activities such as swimming due to the low levels of such activities such among Pakistani and Bangladeshi women in the UK [13]. Strategies to optimise compliance included phone calls or text messages (depending on participant’s indicated preference) mid-way through data collection, a pictorial activity diary with reminders on how to wear the accelerometer, when to put it on and take it off, and a £10 shopping voucher for returning the accelerometer after completion.

### Data reduction and statistical analyses

Accelerometer data were downloaded using Actilife 6 data analysis software (Actigraph, LLC, Pensacola, Florida). A valid day of measurement was defined as at least 600 minutes of registered wear time [32]. Participants with at least 3 valid days of activity were included in this analysis to reflect routine activity and variations in activity [33] At least one weekend day was included in the 3 valid days. Non-wear time was defined as more than 60 successive minutes of zero counts. Data were reduced using Kinesoft software (v3.3.75; Kinesoft, Saskatchewan, Canada) to derive counts per minute (CPM), minutes of light intensity physical activity (LPA), MVPA and ST. Cut points used to determine minutes spent at intensity levels were chosen based on previous research. Cut points were: sedentary <99counts/min [34–36]; light activity 100–1951 counts/min; moderate activity 1952-5724counts/min; vigorous activity 5725-9498/min; and very vigorous activity 9499- ∞/min [37].

Descriptive statistics (means, SDs, percentages) for all variables were calculated. PA/ST data were nonparametric and therefore log transformed for analysis. Independent t-tests or one-way ANOVAs using covariance were conducted to determine differences in PA/ST between age, BMI, body fat percentage, language ability, migration status, waist circumference, and IMD rank. Bonferroni’s post hoc test was used to control for Type I error. The independent effects of potential socio-demographic predictors of PA/ST were explored using multiple linear regression analysis in which all key predictor variables were entered using the enter method. These variables included age, sex, BMI, waist circumference, body fat percentage, IMD rank, literacy, and migration status, which were selected based on a review of the literature and the aims of the study. Pearson correlation coefficients were calculated to determine collinearity between predictor variables. All statistical analyses were conducted using PASW 18.0 (Quarry Bay, Hong Kong).

## Results

Of the 167 SA women recruited to this study, 27 (16%) were excluded due to health reasons (n = 3), dropping out of the study (n = 7), or had incomplete data (n = 17). Of those with incomplete data, 14 were excluded due to lack of complete accelerometer data due to 6 forgetting to wear the accelerometer and 8 failing to wear the device long enough to obtain 3 valid days of data. Overall accelerometer compliance was very good, at 90.91%. The compliance strategies noted earlier may have contributed to this high compliance rate. A total of 140 women with complete demographic, anthropometric, and accelerometer data were included in the final sample.

### Participant characteristics

Age, BMI, waist circumference, migration status, body fat percentage, IMD quintile, and self-reported language ability of participants are shown in Table 1. Over three quarters of the sample was between 18–64.9 years of age. Over 90% (n = 127) of the sample was classified as overweight or obese based on WHO guidelines for BMI categories for SA adults [7], with 67.9% having a waist circumference greater than 80 cm. Although this proportion of the sample who are overweight or obese may appear high, it is important to note that the Health Survey for England, among others, uses European thresholds for BMI, thus underestimating the prevalence of overweight and obesity among SA [38]. If the non-South Asian specific BMI categories for overweight and obesity were applied to the current sample, 25.7% and 35.0% would be defined as overweight or obese, respectively. Most of the sample (80.7%) were categorized as being in the two most socio-economically deprived quintiles based on the Welsh IMD ranking [39]. With regards to migration history, 23.6% (n = 33) of the sample were born in the UK, 35.0% (n = 49) born in Bangladesh and 42.2% (n = 58) born in Pakistan. The majority (69.3%; n = 97) of women reported that they were fluent in English. Approximately 66.5% (n = 93) reported having been diagnosed with a chronic medical condition such as type 2 diabetes, high cholesterol or high blood pressure; 90% (n = 84) of women who reported having a chronic medical condition were currently receiving medical treatment (e.g., medication).

**Table 1:** **Participant characteristics & comparisons of physical activity and sedentary time**

			Mean (SD)	
Age (mean ± SD),yr	46.5 ± 14.3			
Age categories, ***n*** (%)		Sedentary (mean min/day)*	LPA (mean min/day)*	MVPA (mean min/day)*
18-64.9 yrs	106(75.7)	521.94(12.81)	282.30(107.21)^***a***^	39.63(2.87)^***a***^
≥65 yrs	34(24.3)	549.33(14.73)	259.71(103.05)	23.30(4.07)
Migration status**, *n* (%)				
Born within UK	33(23.60)	519.21(87.88)	316.45(106.68)^***b***^	37.72(21.40)
Born in Bangladesh	49(35.01)	523.57(91.95)	288.95(113.83)	33.19(22.11)
Born in Pakistan	58(42.43)	539.34(71.09)	253.42(95.09)	27.54(20.38)
Literacy level**, *n* (%)				
Self-reported fluency in English	97(69.30)	531.76(11.16)	271.92(108.23)	36.39(2.79)
Self-reported non-fluency in English	43(30.70)	522.82(24.11)	274.50(105.13)	28.61(5.52)
BMI (mean ± SD), kg/m^2^	27.8 ± 5.5			
BMI categories**, *n* (%)				
Underweight*** (<18.5)	1(.80)	445.82(45.51)^***c***^	272.04(102.45)^***c***^	43.30(13.32)^***c***^
Normal weight*** (18.5-23)	12(8.60)	406.23(37.05)^***c***^	284.40(101.37)^***c***^	52.89(9.09)^***c***^
Overweight*** (23.1-27.5)	24(17.10)	555.78(17.49)	285.37(119.09)	38.38(4.72)
Obese*** (>27.5)	103(73.50)	533.67(11.50)	266.49(104.64)	31.90(2.95)
Waist circumference (mean ± SD), cm	91.6 ± 14.7			
Waist category, *n* (%)				
Healthy (≤79.9 cm)	45(32.10)	504.21(38.11)	268.15(105.18)	42.23(9.69)
Unhealthy (≥80 cm)	95(67.90)	533.03(10.38)	273.30(108.45)	33.95(2.55)
Body fat(mean ± SD)	41.7 ± 23.1			
IMD Quintiles****, *n* (%)				
1 (most deprived)	52(37.1)	525.71(17.34)	279.11(104.06)	33.20(3.7)
2	61(43.6)	523.11(14.33)	283.24(104.89)	36.45(3.8)
3	27(19.3)	561.60(24.27)	276.07(106.09)	32.16(2.7)
4	0(0)			
5 (least deprived)	0(0)			

*log-transformed data, untransformed means included for all activity data for ease of understanding; **Controlling for age; ***BMI Categories as defined for South Asians by the WHO [9]; ****Based on Index of Multiple Deprivation ranks for Wales [37].

^***a***^significantly (p < 0.05) different from ≥65 yr age group; ^***b***^significantly (p < .001) different from participants born in Bangladesh and Pakistan; ^***c***^significantly (p < .001) different from overweight and obese groups.

### Physical activity levels

Mean ST per day was 530.20 ± 81.76, mean LPA was 39.44 ± 15.16 and mean MVPA per day was 34.66 ± 21.52. Pearson’s correlations showed a positive relationship between LPA and MVPA (*r =* .355, N = 140, p < .001) indicating that as LPA increases so does MVPA. A negative relationship was seen between LPA and ST (*r =* -.610, N = 140, p < .001) indicating that as LPA increases, ST decreases. Similarly, a negative relationship between MVPA and ST was seen (*r =* -.270, N = 140, p < .001), indicating that as MVPA increases, ST decreases.

Just over half, or 53% (n = 74) of the sample met current recommendations for PA of accruing at least 30 minutes of MVPA per day. When MVPA data were examined to determine those accruing at least 30 minutes per day in consecutive 10-minute bouts, the percentage of those meeting recommendations fell to 35% (n = 48). With regards to ST, 64% (n = 89) of the sample spent 5 or more hours of their day being sedentary.

Table 1 shows the results of independent t-tests indicating a significant difference in the amount of time spent in daily LPA (*t =* 2.306, *p <* 0.05) and MVPA (*t =* 3.101, *p <* 0.05) between women in the younger age group (18–64.9 yrs) and older age group (≥65 yrs), with younger women accruing significantly more of both. Interestingly, 35% of women aged 65 years or older met the daily PA recommendation of 30 minutes or more of MVPA most days (23.30 ± 4.07 minutes per day), while 42% women aged 18–64.9 years met the PA recommendation (39.63 ± 2.87 minutes per day). There were no significant differences between age groups for ST.

Table 1 shows the results of ANCOVAs for LPA, MVPA and ST. BMI effects were seen for LPA, MVPA and ST when controlling for age. Mean daily ST was significantly (p < .001) different between normal weight, overweight and obese BMI categories, with normal weight participants having lower ST. There were no significant differences in mean daily ST between participants in the underweight and normal weight categories. The same BMI effect was seen for mean daily LPA and MVPA. Women classified in the normal weight category performed the highest mean daily MVPA (52.89 ± 9.09 minutes). Women born in the UK accumulated significantly more LPA than those born in Bangladesh or Pakistan (p < .001).

Pearson correlation coefficients did not indicate collinearity between the predictor variables included in multiple linear regression analyses. Multiple linear regression analysis using socio-demographic predictor variables resulted in no significant models for ST (Table 2). Table 3 shows the results of significant models for LPA, with one significant model emerging. Age and waist circumference explained 7.3% of the variance in LPA (F(2,138) = 3.955, p < .05). Table 4 shows the results of significant models for MVPA. One significant model also emerged for MPVA, accounting for 14.9% of the variance (F(2,138) = 6.407, p < .01). Again, age and waist circumference were significant predictors.

**Table 2:** **Multiple regression models for sedentary time**

Model	Variable	B	SE(B)	β	t	Adjusted R ^2^	Sig. ( ***p*** ) ^*^
1	Age	0	0.001	0.039	0.197	-0.01	0.47
	BMI	0.00	0.00	0.11	0.63		
	IMD Rank	0.00	0.00	0.02	0.11		
	Body fat %	-0.01	0.00	-0.35	-1.97		
	Waist circumference	0.00	0.00	0.25	1.04		
	Language^+^	-0.02	0.03	-0.12	-0.74		
2	Age	0.00	0.00	0.04	0.22	0.02	0.34
	BMI	0.00	0.00	0.11	0.63		
	Body fat %	-0.01	0.00	-0.35	-2.00		
	Waist circumference	0.00	0.00	0.25	1.10		
	Language^+^	-0.02	0.03	-0.12	-0.77		
3	BMI	0.00	0.00	0.10	0.60	0.04	0.23
	Body fat %	-0.01	0.00	-0.36	-2.17		
	Waist circumference	0.00	0.00	0.28	1.53		
	Language^+^	-0.02	0.03	-0.11	-0.76		
4	Body fat %	-0.01	0.00	-0.36	-2.16	0.05	0.15
	Waist circumference	0.00	0.00	0.33	1.97		
	Language^+^	-0.02	0.03	-0.11	-0.78		
5	Body fat %	-0.01	0.00	-0.33	-2.06	0.06	0.09
	Waist Circumference	0.00	0.00	0.30	1.86		

^+^Language category (Fluent in English or not fluent in English; *Significance level set at p < 0.05.

**Table 3:** **Multiple regression models for light physical activity**

Model	Variable	B	SE(B)	β	t	Adjusted R ^2^	Sig. ( ***p*** ) ^*^
1	Age	-0.002	0.002	-0.167	-1.287	.073	0.02
	Waist circumference	-0.003	0.002	-0.192	-1.478		

*Significance level set at p < 0.05.

**Table 4:** **Multiple regression models for MVPA**

Model	Variable	B	SE(B)	β	t	Adjusted R ^2^	Sig. ( ***p*** ) ^*^
1	Age	-0.008	0.004	-0.317	-1.783	0.149	0.03
	Waist circumference	-0.007	0.007	-0.229	-1.041		

*Significance level set at p < 0.05.

Table 5 shows activity data when investigated by weekdays and weekend days. Mean values for daily ST, daily LPA and daily MVPA were all significantly different. On weekdays participants accrued 24% more MVPA (36.12 ± 12.53) than on weekend days (27.43 ± 14.24). On weekend days women accrued 8% more ST (560.31 ± 146.93) and 12% more LPA (43.03 ± 14.45) than on weekdays (517.77 ± 67.86 and 43.03 ± 14.45).

**Figure 1:**
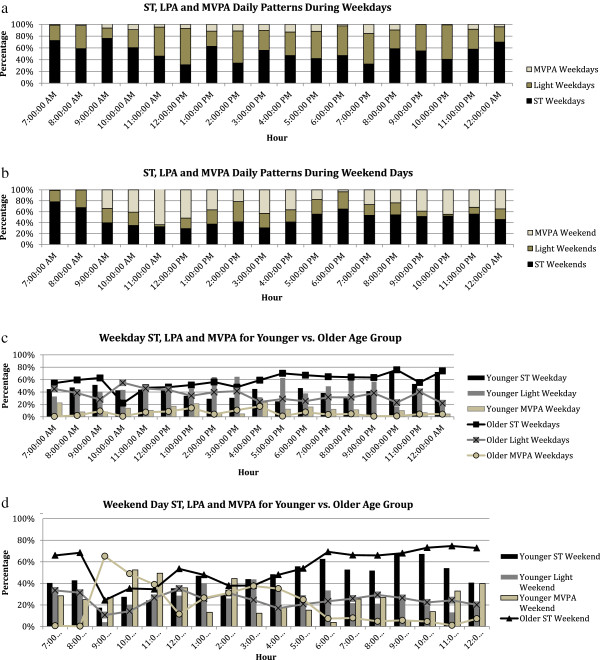
**Weekly physical activity and sedentary time patterns. (a)** Hourly ST(black), LPA(gray) and MVPA(light gray)patterns (% of measured time) for weekdays, **(b)** Hourly ST(black), LPA(gray) and MVPA(light gray) patterns (% of measured time) for weekend, **(c)** comparison of younger (bar) and older (line) age groups for ST, LPA and MVPA on weekdays, **(d)** comparison of younger (bar) and older (line) age groups for ST, LPA and MVPA on weekend days.

Figure 1 shows daily patterns of ST, LPA and MVPA during waking hours. As can been seen in Figure 1a, a higher percentage of each hour during weekdays is spent in ST or LPA for the total sample, while little is spent in MPVA. Amount of time spent in MVPA peaked during the hours of 1 pm-5 pm. On the weekend the percentage of MVPA increased for each hour of the day, with peaks during 9 am-1 pm, 3-4 pm, and 9 pm-midnight (Figure 1b). As MVPA increased during these hours, LPA decreased and ST held relatively constant. Six women spent the hours of 3 am-4 am participating in MVPA (data not shown). When activity patterns were explored by age group, younger women appeared to participate in more MVPA on weekdays than older women, although patterns were similar from 7 pm-midnight in both age groups (Figure 1c). Throughout the weekday younger and older women exhibited similar patterns for LPA, except during the hours of 7 am and 10 am during which older women engaged in more LPA. Younger women spent a lower percentage of time in ST between 12 pm-7 pm when compared to older women, while both age groups spent a similar percentage of time during 8 pm-midnight in ST on weekdays. Conversely, both age groups spent a similar percentage of time in ST on the weekend and showed a similar pattern of ST across the day (Figure 1d). LPA percentages were similar for both age groups across the weekend day (Figure 1d). Older women increased time spent in MPVA on the weekend, especially between the hours of 9 am-11 am and 1 pm-5 pm (Figure 1d). Between the hours of 7 pm-midnight, older women’s MVPA was similarly low on weekdays and weekend days.

**Table 5:** **Weekday vs. weekend physical activity and sedentary time**

Mean (SD)		
	Weekdays	Weekend days
ST (min/day)	517.77(67.86)^***a***^	560.31(146.93)
LPA (min/day)	38.31(16.52)^***a***^	43.03(14.45)
MVPA (min/day)	36.12(12.53)^***a***^	27.43(14.24)

*Note:* One-sample t-tests for log transformed data. Actual means included for clarity of interpretation. ^***a***^significantly different from weekend days (p < .001).

## Discussion

This study has reported the levels and patterns of PA/ST and explored the socio-demographic predictors among a sample of SA women in the UK. Women in our sample were overweight/obese and highly socioeconomically deprived. Based on previous studies on SA women these results are to be expected and support the need for healthy lifestyle interventions among this group [18, 19]. One major finding from this study is the high percentage of SA women who met PA recommendations, with 53% for total MVPA and 35% when examined as consecutive 10-minute bouts of MVPA. These values are higher than would be expected based on studies conducted to date that focused on self-reported PA. The only two additional published studies to objectively measure PA among SA women found similar rates of PA, although measurements were taken with other devices [21, 22]. The non-randomized nature of this study may have resulted in a sample that is particularly active and less sedentary than the general population of SA women in the UK., although more research is needed with comparable measurement instruments and cutpoints. These results are different to the general UK population of women. The Health Survey for England found that 4% of the general population of women in the UK meet PA recommendations [16].

Increasingly LPA is being investigated due to its potential for health benefits particularly in the area of diabetes and CVD [40]. Moreover that it may be easier shifting populations from ST to LPA, or LPA to MVPA than to try to push a very sedentary population to engage in MVPA, which may pose more significant barriers. Our sample of SA women showed relatively high levels of LPA (an average of nearly 40 minutes per day), although comparisons with other samples of SA women and the general population are challenging due to a lack to reporting. It would seem that if it is indeed easier to move from LPA to MVPA, then this group of women has great potential for even more to achieve PA recommendations by increasing the intensity of activity that they already engage in. Additionally, our sample (of all ages) engaged in an average of 5.5 hours of ST per day. Shifting ST to LPA may help to improve the health of SA women and other comparable groups who suffer from high rates of chronic disease morbidity and mortality. Although the HSE found that the general population of UK women engaged in nearly 6 hours of ST per day, slightly more than our sample, the high level of ST in our sample and nationally indicate a need for health promotion and intervention strategies to reduce ST [16].

Age and BMI effects were seen for LPA and MVPA in this sample. As may be expected, older and overweight/obese engaged in significantly less LPA and MVPA than younger and normal or underweight participants. Similar effects have been seen in other studies of SA women as well as in white European groups [18, 41]. Similar BMI effects were seen for LPA and ST, indicating that overweight and obese SA women engage in more ST than normal weight and underweight SA women. The same age effect was not seen for ST, suggesting that age may not be an influencing factor on ST of SA women. This is supported by the lack of significant multiple linear regression models for ST. No significant regression models for ST may indicate a lack of pertinent predictor variables measured in this study. In future studies it is recommended that psychosocial, environmental and cultural factors are measured and included for modeling. Significant multiple regression models for LPA and MVPA indicate that age and waist circumference were the strongest predictors of activity in this sample. The higher the age and waist circumference, the lower the activity levels. Caution should be used when interpreting these results, as although models were significant they explained a very small amount of variance in LPA or MVPA. Again, it is recommended that in future studies, psychosocial, environmental and cultural factors are measured and included for modeling. Of particular importance to a population such as SA women may be emotional and psychological stress, such as that related to migration, discrimination or cultural tensions between minority and majority groups [42].

Investigation of PA/ST patterns on weekdays and weekend days revealed statistically different daily averages for both measures. SA women engaged in more LPA and ST on weekends and more MVPA during weekdays. This change in weekend and weekday activity has been shown in other accelerometer studies on adults [41] although these studies have not investigated ethnic minority PA/ST weekly patterns. Hourly patterns of MVPA indicate that SA women are engaging in higher levels of MVPA before and after meal times during the day. When not engaging in MVPA women are either sedentary or engaging in LPA. Older and younger women engaged in a similar percentage of ST on the weekends, while during the week younger women were less sedentary. This may indicate that younger women have more barriers, fewer reasons or less motivation to engage in PA on weekends. These data also indicate that some participants are active during less common times of day; this likely reflects their extended waking hours, which allow them to accommodate the diverse work schedules of their husbands or other male family members. More research is needed to investigate the reasons for such a drastic change in activity level between weekdays and weekend days, and to provide a more in-depth exploration of patterns of activity throughout a 24-hour period.

## Conclusions

This study offers unique and valuable insights into PA/ST patterns in SA women in the UK, but there are several limitations. Firstly, the cross-sectional and non-randomized design of this study limits generalisability and possibly introduces selection bias. Some determinants, moderators and mediators of PA/ST such as psychosocial, environmental and cultural factors were not assessed. These may be critical to understanding and predicting PA/ST patterns among SA women. The non-randomized nature of this study may have resulted in a sample that is especially active and less sedentary than the general population of SA women in the UK. Although accelerometry is commonly seen as a valid and reliable method of PA data collection, it may suffer from inaccuracy in measuring ST, especially for time spent sitting [43]. Additionally, a more diverse sample of socio-economic backgrounds may yield different results. This study benefits from several valuable strengths. Although it included a relatively small convenience sample, it is the largest sample to date that has objectively measured PA/ST among SA women, a traditionally hard-to-reach ethnic minority group. The use of accelerometry to quantify PA/ST is especially important since self-report measures have been found to be problematic when used with culturally and linguistically diverse groups [44, 45].

This study provides objectively measured PA/ST data and patterns in a cross-sectional sample of SA women in the UK. Data show that over one-third of participants met PA recommendations when MVPA was accumulated in bouts of 10 minutes. This sample of SA women appears to be less sedentary than UK women in general. This has important implications for future interventions aimed that this group. Interventions will need to stress the importance of accumulating MVPA in bouts of 10 minutes or more to obtain health benefits. The high levels of LPA in this sample of SA women indicate that health promotion and intervention programmes may benefit from focusing on shifting sedentary women to more light intensity activities to gain health benefits, or encouraging women who currently engage in LPA to increase the intensity of activities they already engage in to achieve higher levels of MVPA. This approach may be crucial to the success of these programmes, as well as to increasing the health of SA women, since it is known that interventions to increase ST to MVPA are often unsuccessful [46]. Future research with larger, representative samples is needed in order to gain a clearer picture of ST among SA women and the behaviors that they may be engaging in during that time. The addition of psychosocial, environmental and cultural factors would increase our understanding of the influences on PA/ST in this group of ethnic minority women. Finally, this study explored daily patterns of PA/ST by weekday and weekend days. Understanding of these patterns of activity is important to implementing interventions and health promotion strategies among SA women in the UK.
